# Cross-Cultural Adaptation and Validation of “Malocclusion Impact Questionnaire” into Moroccan Arabic

**DOI:** 10.1155/2020/8854922

**Published:** 2020-09-07

**Authors:** Farid Bourzgui, Samir Diouny, Hadam Mkhantar, Zineb Serhier, Mohamed Bennani Othmani

**Affiliations:** ^1^Orthodontics Department, University Hassan II, Faculty of Dentistry, Casablanca, Morocco; ^2^Department of English Studies, University of Chouaib Doukkali El Jadida, B.P. 27, Route Ben Mâachou, 24000 El-Jadida, Morocco; ^3^Private Practice, 6 Ang Rue Mausolé et Tabit Bnou Koraa Etg 3, Appt 5 Rés Sara, Casablanca, Morocco; ^4^Laboratory of Medical Informatics, University Hassan II, Faculty of Medicine& Pharmacy, Casablanca, Morocco

## Abstract

**Introduction:**

The malocclusion index questionnaire (MIQ) is widely used in research and clinical practice. To our knowledge, there are no studies of its use in Moroccan patients.

**Aim:**

The objective of this study was to translate and culturally adapt the malocclusion impact questionnaire (MIQ) into Moroccan Arabic and to assess its reliability and validity among a sample of young Moroccan teenagers. The PIDAQ was cross-culturally adapted into Malay version by forward- and backward-translation processes, followed by psychometric validation.

**Materials and Methods:**

The MIQ was cross-culturally adapted into Moroccan Arabic by forward- and backward-translation processes, followed by psychometric validations. The MIQ was completed by a representative sample of 94 Moroccan adolescents aged 12–17 years selected randomly from five public schools of Casablanca. Internal consistency reliability was determined from Cronbach's alpha, and the intraclass coefficient of the item scores was obtained in 1 month in a subset of 30 subjects. Data were analyzed using the Statistical SPSS software, version 16.0, SPSS Inc, Chicago, IL, USA.

**Results:**

The MIQ was completed twice by each participant at one-month interval to assess test reliability; the intraclass coefficient was *r* = 0.958, showing an excellent reproducibility. The internal consistency demonstrated the reliability of the questionnaire with Cronbach's alpha coefficient of 0.917. The validity of the questionnaire was assessed by evaluation of the relationship between the total scores of the MIQ and normative need for orthodontic treatment according to ICON. The questionnaire showed an insignificant correlation (0.129, *p* < 0.05).

**Conclusion:**

The Moroccan Arabic version of the MIQ was found to be reliable, whereas it has unacceptable validity according to ICON, and hence, it is unlikely to be a useful measure in orthodontic clinical trials for Moroccan adolescents.

## 1. Introduction

Malocclusion is defined as an “anomaly which causes disfigurement or which impedes function, requiring treatment if the disfigurement or functional defect is likely to be an obstacle to the patient's physical or emotional well-being” (WHO) [1]. It is one of the most common oral problems, together with dental caries and periodontal disease. The reasons to develop malocclusion could be due to abnormal size and shape of the teeth, extra abnormal teeth that causes a misalignment, crowding, or displacement of another tooth.

Over the years, several measures have been developed with a view to evaluate orthodontic treatment need, complexity, and outcome. One of these instruments involved the Index of Complexity Outcome and Need (ICON), as developed by Daniels and Richmond [2]. Although its results were promising, the ICON was criticised for not taking into consideration the patient's perception.

Malocclusions are highly prevalent and can result in physical, social, and psychological consequences, thereby exerting a negative impact on quality of life (QoL). It is common for individuals with malocclusion conditions to develop ways to cope with these defects; these strategies involve avoiding smiling, hiding teeth while speaking or laughing, and developing emotional insecurity, fear and social anxiety.

To date, there is a general lack of consensus on how to define and measure QoL. The plurality of definitions and the lack of consensus lead us to think that the term QoL is only understood on a person level or as said by Campbell et al. [3]: “QoL is a vague and ethereal concept, something that many people talk about but nobody knows clearly what it really means.” QoL has been defined by the WHO as “individuals” perception of their position in life in the context of culture and value systems in which they live in relation to their goals, expectations, standards, and concerns” [4].

In fact, oral health-related quality of life (OHRQoL) is recognized by the WHO as an important segment of the global oral health program [5]. Currently, dental research efforts are not only focused on rehabilitating oral-dental diseases but also on exploring the relationship between oral health status and QoL, with a view to evaluate, improve, and maintain it [5].

OHRQoL is “a multidimensional construct that reflects (among other things) people's comfort when eating, sleeping, and engaging in social interaction, their self-esteem, and their satisfaction with respect to their oral health.” [6]. OHRQoL is associated with functional factors, psychological factors (concerning a person's appearance and self-esteem), social factors (interaction with others), and experience of pain or discomfort.

Despite the fact that malocclusion places a significant burden on oral health care provision globally, evidence on impact of malocclusion and orthodontic treatment need on the QoL is conflicting and complex [7]. While some studies [8, 9] argued in favour of a strong relationship between malocclusions and QoL, others have produced conflicting results [10]. This raises the need for the use of OHRQoL indicators specifically developed for malocclusion in conjunction with conventional orthodontic indices useful to assess treatment needs and therapeutic outcomes. Generic indicators are often too long, too complex, and inappropriate for orthodontic patients, often consisting of children and adolescents. Despite these difficulties, three OHRQoL indicators specific to orthodontics were developed:

1. The Orthognathic Quality of Life Questionnaire (OQLQ) by Cunningham et al. [11].
2. The Psychosocial Impact of Dental Aesthetics Questionnaire (PIDAQ) by Klages et al. [12].
3. The Malocclusion Impact Questionnaire (MIQ) by Benson et al. [13].

The research team at Casablanca Department of Dentofacial Orthopaedics carried out the transcultural translation and psychometric validation of the first two measures as indicated above [14, 15]. The MIQ is a condition-specific tool of oral health-related quality of life, intended for use in the longitudinal evaluation of interventions for young people with malocclusion, aged between 10 and 16 years. However, the MIQ has not been tested in young people with a cleft of the lip and/or palate or those who may require surgery to correct abnormalities of the jaws [16].

The purpose of the present study was to translate and culturally adapt the MIQ into Moroccan Arabic and test its reliability and validity for measuring QOL in individuals with malocclusion.

## 2. Materials and Methods

The MIQ was translated and adapted to Moroccan Arabic, following the guidelines for the process of cross-cultural adaptation as outlined by Beaton et al. [17].

Four main steps were followed: the first step in the process of transcultural adaptation consisted of the translation of the original version from English into Moroccan Arabic by two bilingual translators, whose mother tongue was Moroccan Arabic, producing two independent versions. A synthesis of the original questionnaire and both initial Moroccan Arabic translations was performed, resulting in version 1. This version was then submitted to a process of conceptual equivalence by a panel of experts. Two translators who possessed a high native-like proficiency, and were blind to the original version of the MIQ, independently translated version 1 back into English. A comparison was made between the original English version and the back-translated version. Following the expert panel meeting, a single consensus translation of the Moroccan Arabic MIQ was agreed upon.

The preliminary version obtained after the process of transcultural adaptation was used in a pilot study to determine its metric properties of reliability and validity. The Moroccan Arabic version of the MIQ was tested on 10 schoolchildren aged 12–16 years. The pilot test was conducted by the Department of Orthodontics at Casablanca School of Dentistry in Morocco.

Based on the results of the pilot study, the participants did not find any difficulties to understand the different items; thereby, no changes were enforced. The time to fill out the questionnaire was limited to 10–17 min. The pilot test showed that the Arabic Moroccan version of the MIQ exhibited appropriate semantic and conceptual equivalence. The resulting version was then back-translated into English to make sure that the translated version reflected the same item content as the original versions. This step was performed by two other bilingual translators: one of the translators is a resident in orthodontics, Department of Orthodontics, School of Dentistry, Casablanca, Morocco; the other is a PhD student in the same school. It took them one week to translate the Arabic Moroccan version into English. None of the translators took part in the first translation or read the original questionnaire. A review committee included two translators, two back-translators, and an English professor at the Department of English, University of Chouaib Doukkali in El Jadida, Morocco. The consensus was established to assess the semantic and conceptual equivalence of the questions and adapted them for the Arabic Moroccan version of the MIQ. After comparing the original version of the questionnaire with the back-translation version, differences in translation and whether these reflected linguistic inaccuracies or cultural differences were debated with alternative wording suggested when needed. This phase resulted in prefinal version of the Moroccan Arabic translation of the MIQ. The final English version validated by the consensus committee and resulting from the back-translation was then submitted to the developers of the MIQ. Pr. Benson, the principal developer considered that the back-translation reflected the same semantics as the original version.

The MIQ was subsequently tested on 94 Moroccan adolescents aged 12–17 years attending 5 public schools from Casablanca city in January-February 2019. The exclusion criteria comprised children who were not granted parental approval to participate in the study. The questionnaire was filled out at the end of the class and in the presence of the teacher. Ethical clearance was obtained from the Ethics Committee of the Faculty of Dentistry, University of Hassan II University, and all participants and their respective teachers were informed about the aims of the study. Access to schools was granted by the Casablanca Regional Academy of Education and Training. The parental consent and authorization of all students were also obtained.

The participants had twenty minutes to fill out the questionnaire. Different types of data were collected:

- Demographic information: age at the time of the investigation and gender: 1 = male and 2 = female.
- The response format for the two global questions: “generally, how do your teeth bother you?”; “generally, to what extent your teeth affect your life?” was a 5-point severity scale and was scored from 0 = not at all, 1 = a little bit, 2 = a bit more, 3 = a lot, and 4 = very much; MIQ consists of a 3-point severity scale with scores: 0 = do not, 1 = a bit, and 2 = very or a lot for negatively worded questions (“nervous” or “shy”) and 0 = very or a lot, 1 = a bit, and 2 = do not for positively worded questions (“happy” and “good looking”).

To check the reliability of the MIQ, 30 schoolchildren from the same schools filled out the questionnaire a second time a month later. After completing the questionnaire, a bimaxillary dental impression was taken from the 94 samples in order to assess malocclusion using the ICON.

Qualitative variables (gender and normative need for orthodontic treatment) were described by their number (count) and percentages. Quantitative variables (age and score of the MIQ) were described using mean and standard deviation. The response format for the two global questions was a 5-point severity scale and was scored from 0 = “not at all” to 4 = “very much.”

MIQ consists of a 3-point severity scale with scores 0 = “do not,” 1 = “a bit,” and 2 = “very or a lot” for negatively worded questions (nervous and shy) and 0 = “very or a lot,” 1 = “a bit,” and 2 = “do not” for positively worded questions (happy and good looking). The scores for each item were added together to obtain a total score, the minimum score being 0, and maximum score being 34; higher scores indicated poorer OHRQoL.

The data were entered into an Excel spreadsheet. Internal consistency reliability was determined from Cronbach's alpha, and the intraclass coefficient of the item scores was obtained in 1 month in a subset of 30 subjects. The intraclass correlation was used to determine the closeness of the scores obtained by the same individual during the two assessments made 1 month apart. Data were analyzed using the Statistical SPSS software (version 16.0, SPSS Inc; Chicago, IL, USA). For all the statics tests, the significance level was set at *p* ≤ 0.05.

## 3. Results

The study sample included 94 schoolchildren, with a response rate of 100%. The demographic information about the participants (*N* = 94) is shown in Table 1. 54.25% were females, and 45.75% were 43 males. The median age was 15.04 years (SD = 0.89).

Table 2 highlights the numbers and proportions of participants responding to the three collapsed categories for the two global questions. Three quarters (75.52%) of the participants reported that their teeth bothered them little or not at all, and nearly three-quarters claimed that their teeth had little or no effect on their life generally. 15.2% believed their teeth bothered them somewhat generally, and 21.2% believed their teeth had an effect somewhat on their life. 8.1% said their teeth bothered them overall quite a bit or very much, and 5% held that their teeth affected them very much or quite a bit on their life.

Table 3 indicates that 25.53% of the schoolchildren felt very happy about their teeth, while 56.38% claimed that their teeth made them feel a bit happy and 18.08% suggested that they did not feel happy about their teeth. 8.51% of the participants felt good looking; 78.72% thought they felt a bit good looking, while 12.77% believed they did not feel good looking because of their teeth. 14.9% of the participants reported feeling very confident, while nearly three-quarters (74,47%) felt a bit confident, and 10.63% did not feel confident at all. A quarter of the respondents (25.53%) expressed feeling very normal, while 65.96% feeling a bit normal; only 8% of the participants believed that they did not feel normal.

**Figure 1:**
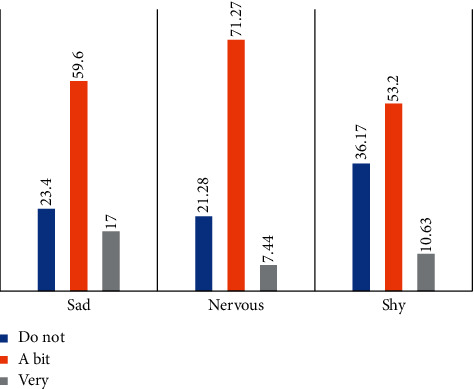
Responses to the MIQ negatively worded questions.

Table 4 gives information about the responses for the rest of MIQ questions. For example, Figure 1 shows that 23.4% of the schoolchildren did not feel sad, while nearly 60% of them felt a bit sad, and only 17% felt sad because of their teeth. 21.28% of the participants believed their teeth did not make them feel nervous, while 71.27% expressed feeling a bit nervous; only 7.44% of the participants believed they felt very nervous because of their teeth.

**Figure 2:**
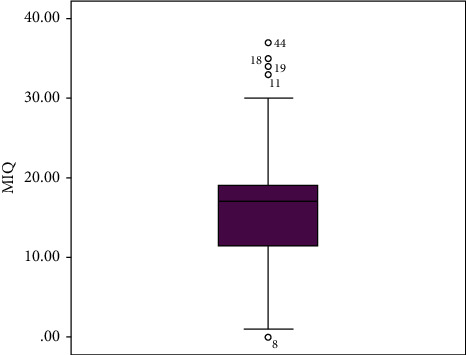
Repartition of the MIQ.

Turning to feeling shy, 36.17% of the participants did not feel shy at all, while 53.2% felt a bit shy and 10.63% expressed feeling very shy.  Figure 2 shows that only one observation had 0 as a score of the MIQ, and 4 observations had higher scores (>30), while the score of the majority of the observations was between 11 and 17.

The Cronbach coefficient of the MIQ was 0.917. The intraclass coefficient correlation of the scores of the responses obtained after administration of the questionnaire twice at a 4-week interval to a sample of 30 same schoolchildren was 0.958 with 95% confidence interval (Table 5).

The convergent validity was evaluated by the association of the MIQ and normative need for orthodontic treatment according to ICON.  Table 6 shows the first component of the ICON: aesthetic (mean = 5.28 and SD = 1.706). 21.3% of the Moroccan participants had a score of 5, followed with 20.2% with a score of 7.

The scores of subjects with an obvious need for treatment were significantly higher (60.63%), while only 39.37% of the participants did not need any orthodontic treatment (Table 7).

The correlation between the MIQ and normative need for orthodontic treatment according to ICON was negative (Table 8).

## 4. Discussion

The malocclusion impact questionnaire (MIQ) was developed and initially validated in the UK. However, till date, this instrument has not been validated in Moroccan Arabic. In fact, this is the first cross-cultural validation of the MIQ reported in the literature. In this study, we describe the adaptation and validation of the MIQ among young children in Morocco and assess its psychometric properties.

MIQ was found to have excellent internal consistency with a score of 0.917. Moreover, reliability in the Moroccan sample was satisfactory, with an intraclass coefficient correlation of 0.958. However, validation using the ICON was found to be insignificant with correlation a score of −0.129.

**Figure 3:**
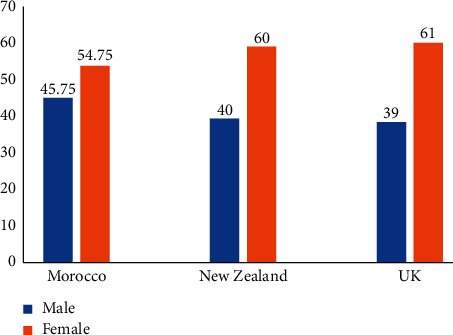
Demographic data: gender for Moroccan, NZ, and UK participants.

Research conducted in the UK [13, 16] and New Zealand (NZ) [18] corroborates the findings of the present study. For example, the ratio of males and females was very similar to the Moroccan, NZ, and UK samples, with a higher proportion of females in all three groups (Figure 3).

**Figure 4:**
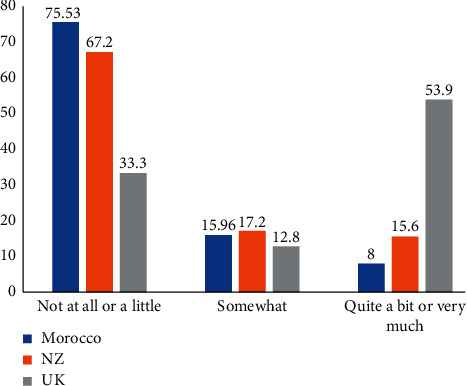
Responses of the Moroccan, NZ, and UK participants to the first global question.

However, some differences in terms of spacing and crowding were worth mentioning. For instance, the Moroccan participants had a higher percentage of spacing in the upper arch compared to the UK samples and NZ samples, who had almost equal proportion. There were also a lower proportion of Moroccan participants with moderate crowding in the upper arch compared to the UK sample (Table 9). Results of the present study suggest that even though the participants performed similarly with young people in New Zealand and the UK, there were some differences between the three samples. The main difference was that Moroccan youngsters were less concerned about their teeth than UK counterparts. According to the responses to the global questions (Figure 4), the majority of Moroccan samples and NZ participants reported that their teeth had a little or no effect on their life overall and that their teeth bothered them a little or not at all.

There was also a higher proportion of UK participants who claimed that their teeth affected them quite a bit or very much on their life overall, and that their teeth bothered them quite a bit or very much compared to the Moroccan and NZ samples.

The Cronbach *α* coefficient for the MIQ, which is a good estimate of internal consistency, was slightly lower in the Moroccan sample (0.917) than in the NZ sample (0.924) but slightly higher than that in the UK sample (0.906). The reliability of the responses was obtained from 30 Moroccan schoolchildren and from 56 UK samples, after a one-month interval was satisfactory, with an intraclass coefficient correlation of 0.958 in Moroccan sample and 0.78 in the UK sample.

The correlations between the global questions and MIQ (overall, how much do your teeth bother you? NZ Spearmen‟ rho was 0.583, overall, how much do your teeth affect your life? UK Spearmen‟ rho = 0.733), and second, the correlation between the total CPQ11-14-ISF16 and the total MIQ scores was (NZ Pearson rho = 0.625, UK rho = 0.751) [18].

The validity of MIQ in the Moroccan sample, however, was performed by evaluating the correlations between the total MIQ scores and normative need for orthodontic treatment according to ICON. Our results showed no significant correlation between the MIQ and ICON (−0.129). The measure of the ICON showed that the majority of the schoolchildren needed orthodontic treatment (60.63%), and compared with the responses to the MIQ questions, the study showed no correlation between the two. Several reasons might explain this noncorrelation.

To begin with, the Moroccan study was carried out in a nonclinical setting, unlike the UK and NZ studies which took part in dental teaching hospitals. This means that both the UK and the NZ participants were aware of their oral problems. Unlike the selection of the UK and the NZ samples, the selection of the Moroccan sample was based on school units and not on individuals; it is widely established that the random selection of schools gives biased sample and wider confidence intervals.

The majority of the Moroccan participants chose safer or neutral answers to the MIQ questions: “a little bit” as an answer to the positively worded questions, as well as the negatively one, whether they needed orthodontic treatment or not (Figure 1).

These answers may be due to the fact that the students did not take the questionnaire seriously, so they filled out the questionnaire as quick as possible, thus distorting the results, or they answered the questions in the way that they believed the researcher wanted them to do so, rather than according to their own perceptions. Moreover, the spirit of rebellion in most teenagers could have a negative effect on the results; they might have given answers contrary to what they really thought.

Another parameter which might influence the results is the family environment in which the participants were raised. Parents might influence the beliefs and the image that teenagers have of themselves. For example, if they keep telling their children “you have a good smile,” they will grow up with some confidence and a high self-esteem even if clinically they needed orthodontic treatment.

During the past decade, several instruments have been developed to assess the impact of oral health on people's lives. Several studies have shown that malocclusion has an effect on the everyday life and activities of young people [19]; however, two systematic reviews suggested that the association was modest [20], while another study involving British children found out that children with malocclusion had no significant impact on QoL [21].

## 5. Conclusion

As a matter of fact, our study showed that the Moroccan version of the MIQ had good psychometric properties, but no correlation with the objective measurement of orthodontic care need measured by ICON was established. The constitution of our sample (nonconsultant population) meant that the adolescents who took part in this study were not aware of orthodontic care and treatment. This may be due to the peripheral role that oral health plays in the Moroccan social and cultural environment.

Clearly, further research is required to confirm the generalisability as well as the ability of the new measure to assess the validity of the MIQ in young people seeking orthodontic treatment in Moroccan dental hospitals.

## Figures and Tables

**Table 1:** Demographic characteristics (age and gender) (*N* = 94).

	*N*	%
*Gender*		
Female	51	54.25
Male	43	45.75

*Age*		
13	3	3.2
14	21	22.3
15	45	47.9
16	19	20.21
17	6	6.4

**Table 2:** Responses of the Moroccan participants (*n* = 94) to the two global questions.

	“Not at all” or “a little”	“Somewhat”	“Quite a bit” or “very much”
Generally, how do your teeth bother you?	71 (75.53%)	15 (15.96%)	8 (8.51%)
Generally, to what extent your teeth affect your life?	68 (73.7%)	21 (21.2)	5 (5.1)

**Table 3:** Responses of the Moroccan participants to the MIQ positively worded questions.

	“Very”	“A bit”	“Do not”
Happy	24 (25.53%)	53 (56.38%)	17 (18.08%)
Good looking	8 (8.51%)	74 (78.72%)	12 (12.77%)
Confident	14 (14.9%)	70 (74.47%)	10 (10.63%)
Normal	24 (25.53%)	62 (65.96%)	8 (8.51%)

**Table 4:** Responses for the rest MIQ questions.

	“Do not”	“A bit”	“Very”
Smile	21 (22.34%)	62 (65.96%)	11 (11.7%)
Laugh	20 (21.27%)	65 (69.15%)	9 (9.57%)
Seeing photographs	21 (22.34%)	60 (63.83%)	13 (13.83%)
Talking in public	38 (40.42%)	53 (56.38%)	5 (5.32%)
Others having nicer teeth	40 (42.55%)	43 (45.74%)	11 (11.71%)
Being bullied	49 (52.12%)	40 (42.55%)	5 (5.33%)
Making friends	43 (45.74%)	48 (51.06%)	3 (3.19%)
Fitting in with friends	1 (1.06%)	44 (46.8%)	49 (52.12%)
Cover with hand	28 (29.79%)	62 (65.96%)	4 (4.25%)
Biting some food	41 (43.61%)	46 (48.93%)	7 (7.46%)

**Table 5:** Reliability of the Moroccan Arabic version of the MIQ.

	Reliability (internal consistency Cronbach *α* coefficient)	Reproducibility (intraclass correlation coefficient)
Questionnaire	0.917	0.958

**Table 6:** Scores of the aesthetic: ICON.

Aesthetics	*N* (*N* = 94)	%
1	1	1.1
2	2	2.1
3	13	13.8
4	16	17
5	20	21.3
6	16	17
7	19	20.2
8	4	4.3
9	3	3.2

**Table 7:** Distribution of the percentages of the participants to the Moroccan Arabic version of the MIQ according to the orthodontic treatment need as assessed by the index of complexity, outcome, and need (ICON).

Normative need for treatment according to ICON		
	*N*	%
(i) Definite need	57	60.63
(ii) No need	37	39.37

**Table 8:** Correlation between the different scores of the MIQ and the one of the ICONs.

	MIQ	ICON	*P*
Mean	16	52.12	
SD	7.49	19.805	−0.129°
Maximum	37	97	
Minimum	00	14	

**Table 9:** Demographic and occlusal data for Moroccan participants (*N* = 94), NZ participants (*N* = 66), and UK participants (*N* = 184).

Demographic and occlusal data	Morocco (*N* = 94)	New Zealand (*N* = 66)	UK (*N* = 184)
*Gender*			
Male	43 (45.75%)	26 (40%)	71 (39%)
Female	51 (54.25%)	39 (60%)	113 (61%)

*Age*			
10		9 (14.1%)	11 (6%)
11		15 (23.4%)	21 (11.4%)
12		12 (18.8%)	40 (21.7%)
13	3 (3.2%)	12 (18.8%)	44 (23.9%)
14	21 (22.3%)	8 (12.5%)	43 (23.4%)
15	45 (47.9%)	5 (7.8%)	23 (12.5%)
16	19 (20.2%)	3 (4.7%)	2 (1.1%)
17	6 (6.4%)		

*Upper arch*			
Spaced	32 (34%)	15 (22.7%)	43 (23.4%)
No crowding or mild (0–4 mm)	30 (31.9%)	41 (62.1%)	50 (27.2%)
Moderate crowding (5–8 mm)	15 (16%)	8 (12.1%)	52 (28.3%)
Severe (>8 mm)	17 (18.1%)	2 (3%)	39 (21.2%)

## Data Availability

The data and all information concerning this study will be provided by the corresponding author.
